# Supplementary figures and images for: TRIM72 restricts lyssavirus infection by inducing K48-linked ubiquitination and proteasome degradation of the matrix protein
Source: PLoS Pathog. 2024 Feb 26;20(2):e1011718. doi: 10.1371/journal.ppat.1011718 (PMC10919858; doi:10.1371/journal.ppat.1011718)

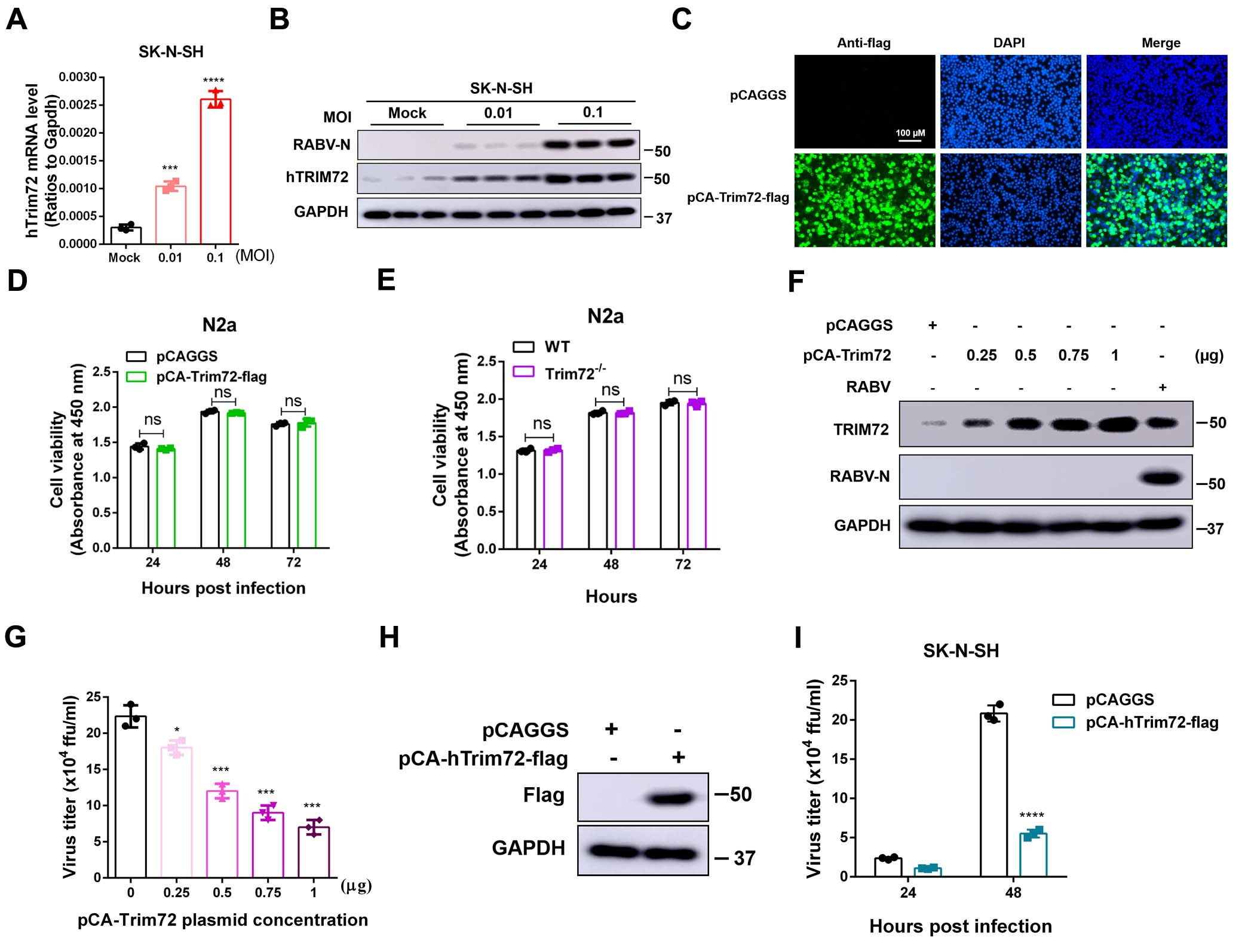

Supplement: S1 Fig — (A-B) SK-N-SH cells were infected with RABV at different MOI for 36 h. The mRNA level of TRIM72 was analyzed by qPCR (A), and the protein levels of TRIM72 and RABV-N were analyzed by western blotting (B). (C) Empty vector (pCAGGS) or TRIM72-flag over-expression vector (pCA-Trim72-flag) were transfected into N2a cells respectively for 48 h, then TRIM72-flag expression level and transfection efficiency were analyzed by indirect immunofluorescence with anti-flag antibody. Scale bar, 100 μM. (D) pCAGGS or pCA-TRIM72-flag were transfected into N2a cells respectively for the indicated time, then cell viability was analyzed. (E) The cell viability of WT and Trim72-/- N2a cells were analyzed at different time points. (F) The protein level of TRIM72 in N2a cells which were transfected with different concentrations of pCA-Trim72 or infected with RABV (MOI = 1) for 48 h were analyzed by western blotting with anti-TRIM72 antibody. (G) Empty vectors or different concentrations of TRIM72 over-expression vectors were transfected into N2a cells respectively for 12 h, then infected with RABV (MOI = 0.01) for 48 h, and the viral titers in the supernatant were analyzed. (H) Empty vector (pCAGGS) or hTRIM72-flag over-expression vector (pCA-hTrim72-flag) were transfected into SK-N-SH cells respectively for 48 h, then hTRIM72-flag level was analyzed by western blotting. (I) Empty vector or hTRIM72-flag over-expression vectors were transfected into SK-N-SH cells respectively for 12 h, then infected with RABV (MOI = 0.01) for the indicated time, and the viral titers in the supernatant were analyzed. Statistical analysis of grouped comparisons was carried out by student’s t-test (*P < 0.05; **P<0.01; ***P<0.001; ****P<0.0001). The bar graph represents means ± SD, n = 3. Western blot data are representative of at least two independent experiments. (TIF) [file ppat.1011718.s001.tif]

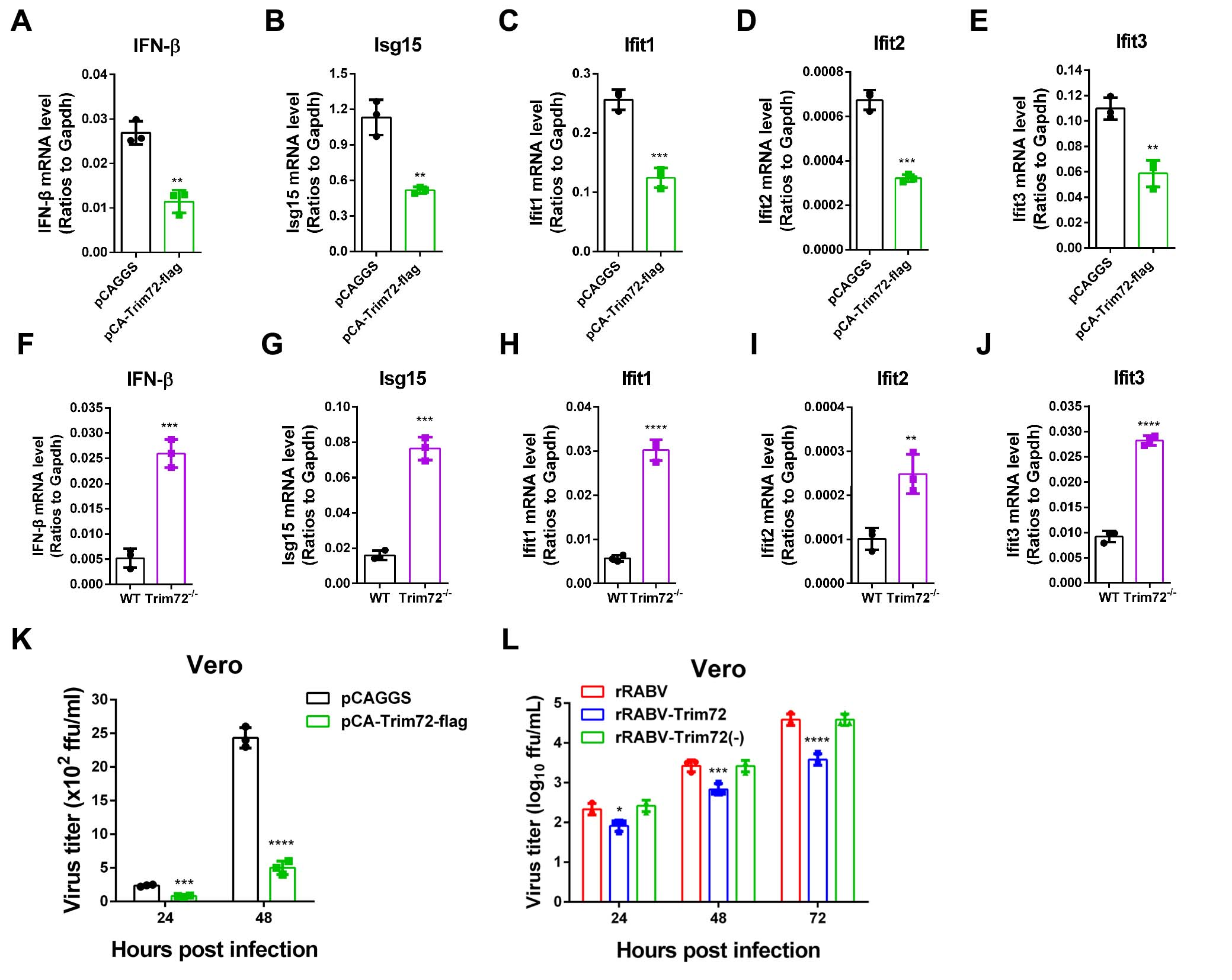

Supplement: S2 Fig — (A-E) Empty vector or TRIM72-flag over-expression vectors were transfected into N2a cells respectively for 12 h, then infected with RABV (MOI = 1) for 48 h, and the mRNA levels of IFN-β (A), Isg15 (B), Ifit1 (C) Ifit2 (D) and Ifit3 (E) were analyzed by qPCR. (F-J) WT and Trim72-/- N2a cells were infected with RABV (MOI = 1) for 48 h, and the mRNA levels of IFN-β (A), Isg15 (B), Ifit1 (C) Ifit2 (D) and Ifit3 (E) were analyzed by qPCR. (K) Empty vector or TRIM72-flag over-expression vectors were transfected into Vero cells respectively for 12 h, then infected with RABV (MOI = 0.01) for the indicated time, and the viral titers in the supernatant were analyzed. (L) Vero cells were infected with different types of rRABVs (MOI = 0.01) and their growth kinetics were compared. Statistical analysis of grouped comparisons was carried out by student’s t-test (*P < 0.05; **P<0.01; ***P<0.001; ****P<0.0001). The bar graph represents means ± SD, n = 3. (TIF) [file ppat.1011718.s002.tif]

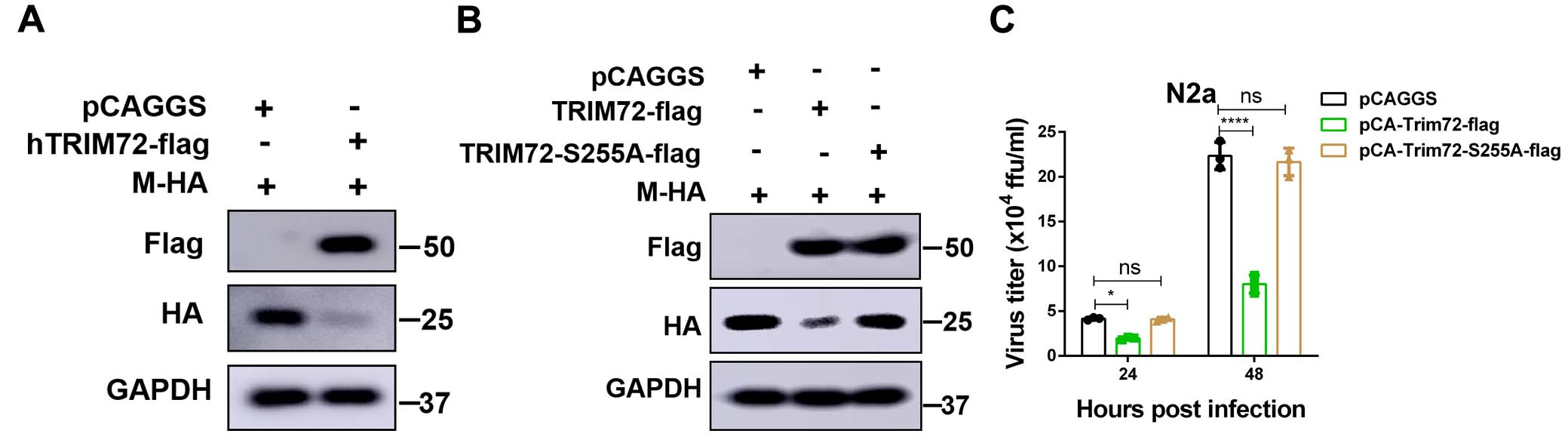

Supplement: S3 Fig — (A) pCAGGS or pCA-hTrim72-flag together with pCA-M-HA were co-transfected into SK-N-SH cells respectively for 48 h, and the protein levels of M-HA and hTRIM72-flag were analyzed by western blotting. (B) pCAGGS, pCA-Trim72-flag or pCA-Trim72-S255A-flag together with pCA-M-HA were co-transfected into N2a cells respectively for 48 h, and the protein levels of M-HA, TRIM72-flag or TRIM72-S255A-flag were analyzed by western blotting. (C) pCAGGS, pCA-Trim72-flag or pCA-Trim72-S255A-flag were transfected into N2a cells respectively for 12 h, then infected with RABV (MOI = 0.01) for 48 h, and the viral titers in the supernatant were analyzed. Statistical analysis of grouped comparisons was carried out by student’s t-test (**P<0.01; ****P<0.0001). The bar graph represents means ± SD, n = 3. Western blot data are representative of at least two independent experiments. (TIF) [file ppat.1011718.s003.tif]

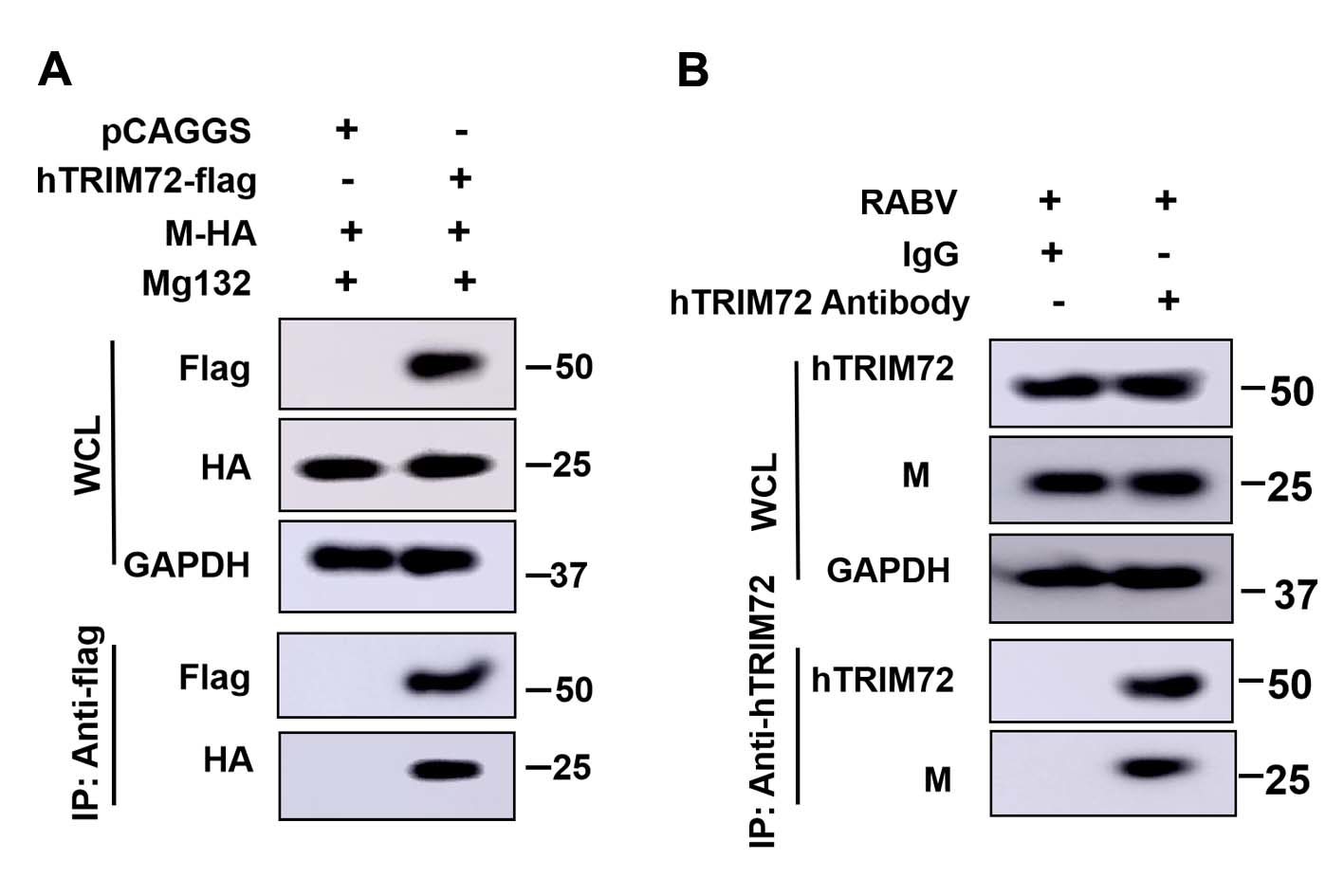

Supplement: S4 Fig — (A) pCAGGS or pCA-hTrim72-flag together with pCA-M-HA were co-transfected into SK-N-SH cells respectively. Then Mg132 (10 μM) was treated, and Co-IP assays were performed with anti-flag antibody post-transfection for 48 h. The protein levels of TRIM72-flag and M-HA were analyzed by western blotting. (B) SK-N-SH cells were infected with RABV (MOI = 1) for 48 h, then Co-IP assays were performed with an anti-hTRIM72 antibody and protein levels of hTRIM72 and RABV-M were analyzed by western blotting. Western blot data are representative of at least two independent experiments. (TIF) [file ppat.1011718.s004.tif]

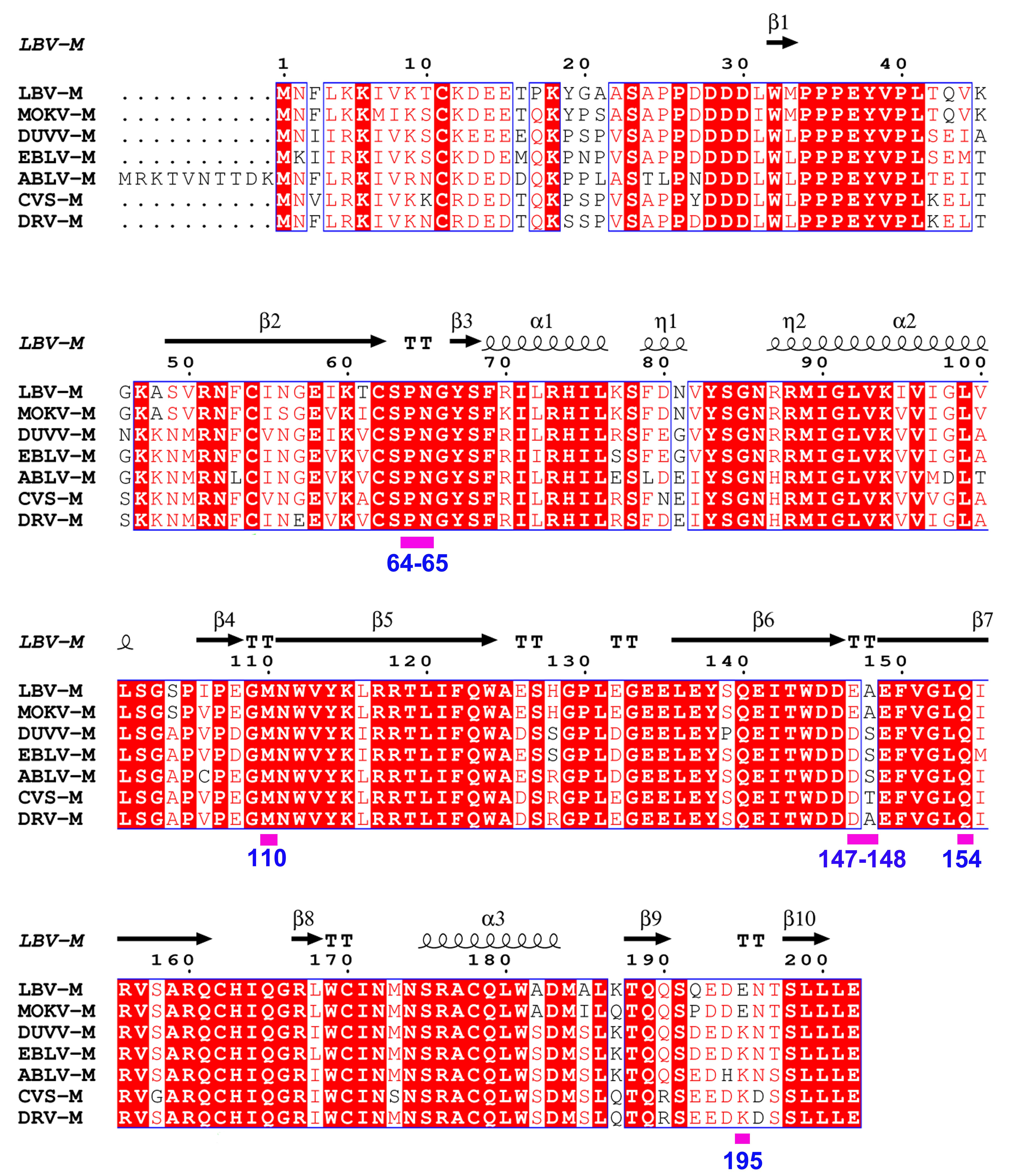

Supplement: S5 Fig — The protein sequences of lyssavirus M were compared and analyzed with ESPript 3.0 online software (https://espript.ibcp.fr/ESPript/cgi-bin/ESPript.cgi). (TIF) [file ppat.1011718.s005.tif]

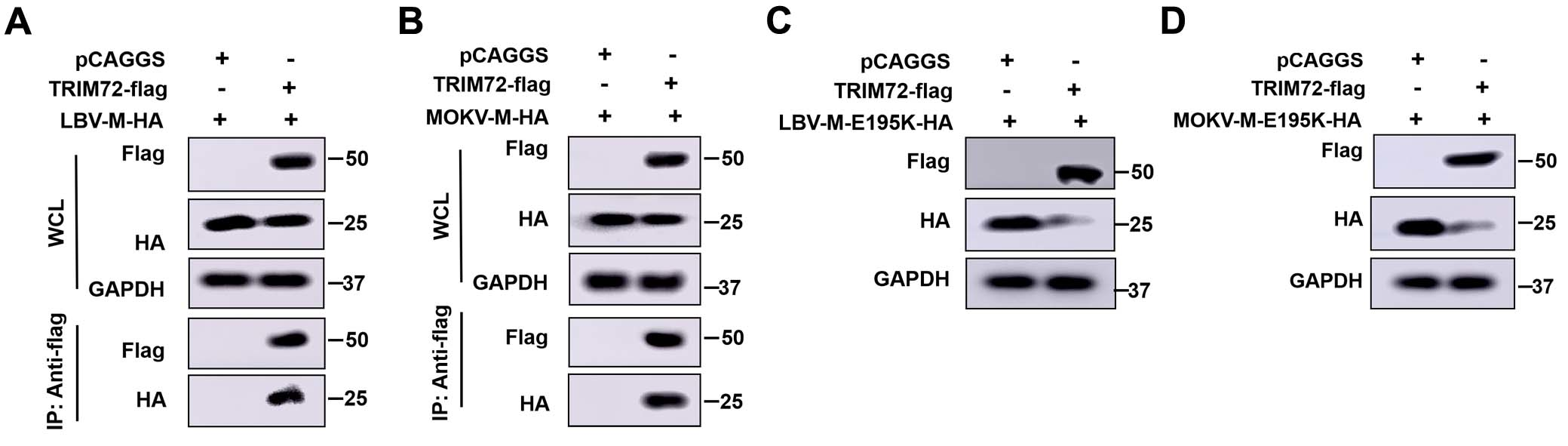

Supplement: S6 Fig — (A-B) HA-tagged lyssavirus M proteins (LBV-M-HA and MOKV-MHA) together with empty vector or TRIM72-flag were co-overexpressed in N2a cells respectively. Co-IP assays were performed with anti-flag antibody post-transfection for 48 h and protein levels of LBV-M-HA (A), and MOKV-M-HA (B), were analyzed by western blotting. (C-D) pCA-LBV-M-E195K-HA or pCA-MOKV-M-E195K-HA together with empty vector or TRIM72-flag were co-overexpressed in N2a cells for 48 h. The protein levels of LBV-M-E195K-HA (C) or MOKV-M-E195K-HA (D) were analyzed by western blotting. Western blot data are representative of at least two independent experiments. (TIF) [file ppat.1011718.s006.tif]
